# Assaying *Paenibacillus alvei* CsaB-Catalysed Ketalpyruvyltransfer to Saccharides by Measurement of Phosphate Release

**DOI:** 10.3390/biom11111732

**Published:** 2021-11-20

**Authors:** Fiona F. Hager-Mair, Cordula Stefanović, Charlie Lim, Katharina Webhofer, Simon Krauter, Markus Blaukopf, Roland Ludwig, Paul Kosma, Christina Schäffer

**Affiliations:** 1 *NanoGlycobiology* Unit, Department of NanoBiotechnology, Universität für Bodenkultur Wien, 1190 Vienna, Austria; fiona.hager@boku.ac.at (F.F.H.-M.); cordula.stefanovic@boku.ac.at (C.S.); 2 Department of Chemistry, Institute of Organic Chemistry, Universität für Bodenkultur Wien, 1190 Vienna, Austria; charlie.lim@boku.ac.at (C.L.); katharina.webhofer.20@ucl.ac.uk (K.W.); simon@krauter.at (S.K.); markus.blaukopf@boku.ac.at (M.B.); paul.kosma@boku.ac.at (P.K.); 3 Biocatalysis and Biosensing Laboratory, Department of Food Science and Technology, Universität für Bodenkultur Wien, 1190 Vienna, Austria; roland.ludwig@boku.ac.at

**Keywords:** cell wall glycopolymer, enzyme assay, kinetic constants, pyruvyltransferase, substrate synthesis

## Abstract

Ketalpyruvyltransferases belong to a widespread but little investigated class of enzymes, which utilise phosphoenolpyruvate (PEP) for the pyruvylation of saccharides. Pyruvylated saccharides play pivotal biological roles, ranging from protein binding to virulence. Limiting factors for the characterisation of ketalpyruvyltransferases are the availability of cognate acceptor substrates and a straightforward enzyme assay. We report on a fast ketalpyruvyltransferase assay based on the colorimetric detection of phosphate released during pyruvyltransfer from PEP onto the acceptor via complexation with Malachite Green and molybdate. To optimise the assay for the model 4,6-ketalpyruvyl::ManNAc-transferase CsaB from *Paenibacillus alvei*, a β-d-ManNAc-α-d-GlcNAc-diphosphoryl-11-phenoxyundecyl acceptor mimicking an intermediate of the bacterium’s cell wall glycopolymer biosynthesis pathway, upon which CsaB is naturally active, was produced chemo-enzymatically and used together with recombinant CsaB. Optimal assay conditions were 5 min reaction time at 37 °C and pH 7.5, followed by colour development for 1 h at 37 °C and measurement of absorbance at 620 nm. The structure of the generated pyruvylated product was confirmed by NMR spectroscopy. Using the established assay, the first kinetic constants of a 4,6-ketalpyuvyl::ManNAc-transferase could be determined; upon variation of the acceptor and PEP concentrations, a *K*_M, PEP_ of 19.50 ± 3.50 µM and *k*_cat, PEP_ of 0.21 ± 0.01 s^−1^ as well as a *K*_M, Acceptor_ of 258 ± 38 µM and a *k*_cat, Acceptor_ of 0.15 ± 0.01 s^−1^ were revealed. *P. alvei* CsaB was inactive on synthetic *p*NP-β-d-ManNAc and β-d-ManNAc-β-d-GlcNAc-1-*O*Me, supporting the necessity of a complex acceptor substrate.

## 1. Introduction

Pyruvate-ketal modified (henceforth termed “pyruvylated”) glycans are found in various kingdoms of life where they have a wide repertoire of biological roles [1]. Pyruvylated galactose, for instance, is an epitope of the *N*-glycans of *Schizosaccharomyces pombe* [2], in the exopolysaccharide (EPS) of *Xanthomonas campestris* [3], and the capsular polysaccharides (CPS) of *Bacteroides fragilis* [4] and *Streptococcus pneumoniae* [5]. Pyruvylated *N*-acetylmannosamine (pyr-ManNAc) is present in peptidoglycan-linked cell wall glycopolymers (CWGPs) of Gram-positive bacteria, where it is an indispensable cell wall ligand for S-layer homology (SLH) domain-containing proteins [6,7]. Prominent examples are the CWGPs of *Bacillus anthracis* and *Paenibacillus alvei* [7,8,9,10], where pyr-ManNAc is found as a single epitope [9] and in a repetitive fashion [6,11], respectively. Furthermore, pyruvylated glycoconjugates are immunostimulatory effectors [2,4,12] and contributors to virulence, exemplified with *Bacillus cereus* [13] and *B. anthracis* [9]. Pyruvylated CWGPs are of importance in the context of anthrax disease, and pyruvylated *Xanthomonas* spp. EPS is required for successful colonisation and pathogenesis in plants [3].

In the NCBI database, there are currently ~9000 protein entries predicted to possess a PS_pyruv_trans (polysaccharide-pyruvyltransferase; PF04230) PFAM domain characteristic of pyruvyl::saccharide-transferases. They commonly require phosphoenolpyruvate (PEP) as a donor substrate and release free phosphate upon pyruvyltransfer to a dedicated acceptor [14]. The best-known example of a pyruvyl::saccharide-transferase in nature is the UDP-*N*-acetylglucosamine-3-*O*-enol-pyruvyltransferase MurA occurring at the early stage of bacterial cell wall peptidoglycan biosynthesis [14]. Despite their documented biological importance, ketalpyruvyltransferases remain a poorly investigated class of enzymes, mainly due to the unavailability of a straightforward activity assay and of suitable acceptor substrates.

Currently available ketalpyruvyltransferase assays depend on on-column product purification and identification, and on labelled and, frequently, custom-made acceptor substrates. Furthermore, they are time-consuming and not suitable for screening purposes (reviewed by Hager et al. [1]). HPLC-based methods have been developed for distinct enzymes of interest, such as Pvgp1 from *S. pombe*, for which pyruvyltransfer onto *para*-nitrophenyl-galactoside was monitored at 265 nm [15] and WcfO from *B. fragilis*, for which pyruvylation of a synthetic, fluorescent, polyisoprenoid CPS biosynthesis intermediate mimic (2-cyanophenylamino-undecaprenyldiphosphate-2-acetamido-4-amino-2,4,6-trideoxy-galactosyl-galactose) was monitored by fluorescence detection [4].

Recently, we identified CsaB from the *P. alvei* CWGP biosynthesis pathway as the enzyme catalysing ketalpyruvyl-transfer onto the ManNAc residue of the [→4)-β-d-GlcNAc-(1→3)-β-d-ManNAc-(1→] CWGP disaccharide repeats, using a β-d-ManNAc-(1→4)-α-d-GlcNAc-diphosphoryl-phenoxyundecyl (ManNAc-GlcNAc-PP-UndPh) acceptor precursor analogue [11]. In a one-pot reaction, chemically synthesised GlcNAc-PP-UndPh was enzymatically elongated to the disaccharide state and pyruvylated by CsaB, followed by product purification and identification by NMR spectroscopy [11]. While the activity of the *P. alvei* CsaB enzyme on ManNAc-GlcNAc-PP-UndPh is a strong indication of ketalpyruvylation taking place at the stage of the lipid-linked disaccharide precursor within this bacterium’s CWGP biosynthesis pathway, other potential acceptor substrates still need to be tested to obtain insight into the substrate range of the enzyme and, importantly, the overall CWGP biosynthesis mechanism. It is currently unclear along which of the two principle pathways of glycoconjugate biosynthesis this CWGP is synthesised—along the Wzy polymerase-dependent pathway, implicating the polymerisation of individual repeating units from their lipid-linked precursors at the extracytoplasmic space, or along the ABC transporter pathway, implicating the export of the full-length CWGP [16]. Notably, in line with our previous data on *P. alvei* CsaB activity, for *B. anthracis* it was proposed that CsaB modifies undecaprenyl-pyrophosphate-linked CWGP trisaccharide repeats with pyruvate-ketal, galactosyl and acetyl groups prior to transfer across the cytoplasmic membrane by a Wzx-like protein, followed by the polymerisation of the fully modified repeats by a Wzy-like protein at the exterior side [17].

In this study, the Malachite Green Phosphate Assay [18,19,20,21] was established as a fast method to assay *P. alvei* CsaB-catalysed ketalpyruvyl-transfer from PEP to different sugar acceptor substrates, circumventing tedious reaction product purification and allowing the determination of kinetic constants. The principle of the assay is the complexation of free orthophosphate that is released from PEP during enzymatic pyruvyltransfer with the Malachite Green dye and molybdate, yielding a coloured phosphomolybdate complex allowing the quantification of the reaction. In this study, the assay was evaluated using a newly synthesised and pure preparation of the proven *P. alvei* CsaB acceptor ManNAc-GlcNAc-PP-UndPh. To learn about the substrate specificity of the CsaB enzyme, synthesised *para*-nitrophenyl-ManNAc and a methyl-glycoside were also tested as acceptors. The CsaB enzyme was produced as recombinant hexahistidine-tagged protein in *E. coli* and the formation of the pyruvylated product was confirmed by NMR spectroscopy. The kinetic constants of *P. alvei* CsaB under optimal reaction conditions—with regard to pH, temperature, time and concentration of Mg^2+^—including *V*_max_ as well as *K*_M_ and *k*_cat_ values against the donor and the acceptor substrate were determined.

## 2. Materials and Methods

### 2.1. Analytics of Substrates and CsaB Reaction Products

NMR spectra were recorded with a Bruker Avance III 600 instrument (600.22 MHz for ^1^H, 150.93 MHz for ^13^C, 242.9 MHz for ^31^P) or a Bruker Avance 300 instrument using standard Bruker NMR software. ^1^H spectra were 3.34 (MeOD) and 0.00 (D_2_O, external calibration to 2,2-dimethyl-2-silapentane-5-sulfonic acid) ppm unless stated otherwise. ^13^C NMR spectra were referenced to 49.00 (MeOD) and 67.40 (D_2_O, external calibration to 1,4-dioxane) ppm. ^31^P NMR spectra were referenced to external ortho-phosphoric acid (δ 0.0) for solutions in D_2_O. Structure assignments were based on COSY, HSQC, HMBC and TOCSY data.

### 2.2. Cloning, Heterologous Expression and Purification of Enzymes

*E. coli* cells (Invitrogen, Waltham, MA, USA) were cultivated in Luria–Bertani broth with 100 µg mL^−1^ ampicillin at 37 °C and 180 rpm. The *wecB* gene encoding a UDP-GlcNAc-2-epimerase was amplified with the primer pair wecB_for_NcoI TGCACCATGGCGGTGAAAGTACTGACTGTATTTGGTACG/wecB_rev_XhoI TGACCTCGAGTAGTGATATCCGATTATTTTT*TAA*CGC (Thermofisher Scientific, Waltham, MA, USA; restriction sites underlined; stop codon italicised) from genomic DNA of *E. coli* BL21 [22] by use of Phusion High-Fidelity DNA Polymerase (Thermofisher Scientific). The 1131-bp amplification product was digested with NdeI and XhoI (Fermentas, Waltham, MA, USA) and cloned into NdeI/XhoI-linearised pET28a vector (Novagen). The construct was chemically transformed into *E. coli* DH5α cells for the amplification of plasmid DNA. Transformants were screened by colony PCR using the RedTaq ReadyMix PCR mix (Sigma-Aldrich, St. Louis, MO, USA) and confirmed by restriction mapping and sequencing (Microsynth). Plasmid DNA was isolated from transformed *E. coli* DH5α cells using the GeneJET™ Plasmid Miniprep Kit (Fermentas) and heat-shock-transformed into *E. coli* BL21 (DE3) cells. WecB expression was induced with 0.6 mM isopropyl-β-d-thiogalactopyranoside. Cells were harvested after 4-h incubation at 37 °C/180 rpm (5500 g, 20 min) and the pellet was lysed by sonication (Branson Ultrasonics Sonifier™, Brookfield, CT, USA) and washed with 25 mM sodium phosphate buffer, pH 7.5. Recombinant WecB (rWecB) was enriched using Amicon filters (Merck Millipore Amicon™ Ultra; Merck, Burlington, VT, USA) and purified by size exclusion chromatography (SEC) on a Superdex 200 16/60 column connected to an FPLC system (Biorad, Hercules, CA, USA) using 50 mM Na-phosphate buffer, pH 7.5, as eluent. Fractions of 5 mL were collected and detection was performed at 280 nm.

The pyruvyltransferase CsaB and the UDP-ManNAc transferase TagA from *P. alvei* were produced as tagged proteins in *E. coli* BL21 (DE3) cells using the expression plasmids pET22b_csaB and pMAL_tagA, respectively [11], yielding C-terminally His_6_-tagged CsaB and a maltose binding protein (MBP)-TagA chimera. Recombinant CsaB (rCsaB) and recombinant TagA (rTagA) were purified as described previously [11].

The recombinant enzymes were analysed by 10% SDS-PAGE in a Miniprotean™ apparatus (Biorad Hercules, CA, USA) according to Laemmli [23] upon Coomassie Brilliant Blue G250 (CBB) staining. The protein concentration was determined spectrophotometrically using the protein-specific extinction coefficient and molecular weight obtained from the exPASy ProtParam tool (http://web.expasy.org/protparam)(accessed from 1 March 2020 through 30 June 2021).

### 2.3. Chemical Synthesis and Purification of UDP-α-d-ManNAc

Chemical synthesis and purification of UDP-α-d-ManNAc followed an established route [11].

### 2.4. Chemical Synthesis and Purification of α-d-GlcNAc-Diphosphoryl-11-Phenoxyundecyl (1)

α-d-GlcNAc-diphosphoryl-11-phenoxyundecyl (α-d-GlcNAc-PP-UndPh) (1) was synthesised according to a published protocol [24], followed by an additional purification step after the final deprotection step. Purification was performed by hydrophobic interaction liquid chromatography (HILIC) on a preparative HPLC system (Interchim 4125 with ELSD) with a semi-preparative SeQuant ZIC-HILIC column (250 × 10 mm; VWR) attached to an Interchim 4125 ELSD system with a drift tube temperature of 60 °C, employing a gradient from 100% acetonitrile (ACN)/5% 5 mM NH_4_OAc to 40% ACN over ten column volumes at a flow rate of 7 mL min^−1^. Fractions were lyophilised (Labconco Refrigerated Centrivap Concentrator; Labonco, Kanasas City, MO, USA) and individually checked by ^1^H and ^31^P NMR spectroscopy. Pure fractions, which were in agreement with reported data [24], were combined, lyophilised and used in further enzymatic conversions.

### 2.5. Enzymatic Preparation and Purification of β-d-ManNAc-(1→4)-α-d-GlcNAc-PP-UndPh (2) from (1)

To obtain the β-d-ManNAc-(1→4)-α-d-GlcNAc-diphosphoryl-11-phenoxyundecyl (ManNAc-GlcNAc-PP-UndPh) (2) acceptor, (1) was reacted with the ManNAc-transferase rTagA, either in combination with the UDP-GlcNAc-2-epimerase rWecB and UDP-GlcNAc (Sigma-Aldrich, St. Louis, MO, USA) to produce UDP-α-ManNAc in situ (strategy A), or by direct provision of chemically synthesised UDP-α-ManNAc substrate (strategy B). The procedure was performed according to Hager et al. with minor modifications [11]. Briefly, for strategy A, 4 mM of (1) was incubated with 11.5 mM UDP-GlcNAc (Sigma-Aldrich, St. Louis, MO, USA), 42 µg rWecB and 160 µg rTagA for 1 h at 37 °C in a total volume of 1175 µL of 25 mM sodium phosphate buffer, pH 7.5, containing 10 mM MgCl_2_, which is required for WecB activity. For strategy B, 0.6 mM (1) and 1.5 mM UDP-ManNAc were incubated with 0.3 µg of rTagA dissolved in 20 mM Tris-HCl, pH 7.5, containing 23 mM sodium phosphate without MgCl_2_, for 1 h at 37 °C in a total volume of 1.5 mL.

Following the 1-h incubation at 37 °C, the reactions were incubated overnight at 25 °C to reach completeness. After stopping the reactions with 500 µL of ice-cold dH_2_O, the mixtures were loaded on a (C18) Sep-Pak classic cartridge (Waters; 36 mg sorbens) for selective binding of the lipid-like portion on the acceptor substrate. The rTagA reaction product (2) was eluted with 3 mL of MeOH (collecting 1.5-mL fractions in Eppendorf tubes), after prior removal of unbound material with 5 mL of dH_2_O. The purity of (2) after Sep-Pak purification was checked by ^1^H NMR spectroscopy. For resalting of (2) from the Tris to the sodium form, 150 mg of Dowex 50XW8 (Na^+^) cation exchange resin (VWR) was added per Eppendorf tube, and the tubes were shaken for 2 min and centrifuged (5000 rpm, 2 min). The supernatant, which was of neutral pH, was transferred into a Falcon tube (15 mL). The residual resin was washed three more times with ddH_2_O (2 mL, each), and all supernatants were combined, lyophilised and checked by ^1^H NMR.

### 2.6. Chemical Synthesis of β-d-ManNAc-(1→4)-β-d-GlcNAc-1-OMe (3)

The chemical synthesis of β-d-ManNAc-(1→4)-β-d-GlcNAc-1-OMe (3) as a potential acceptor substrate for the *P. alvei* CsaB enzyme is described in the Supplementary Materials.

### 2.7. CsaB Activity Assay: Mode of Measurement

The Malachite Green Phosphate Assay Kit (Sigma-Aldrich, Cat No MAK307-1KT) is based on the colorimetric quantification of the green complex formed between Malachite Green, molybdate and free orthophosphate by measuring the absorbance value between 600 nm and 660 nm, according to the manufacturer’s instructions.

To optimise the assay for the *P. alvei* pyruvyltransferase CsaB, which requires a non-commercial, difficult-to-access acceptor substrate (i.e., compound (2) from above) in conjunction with PEP as a donor, first, key assay parameters were evaluated using 5 µM NaH_2_PO_4_ in dH_2_O as a test substance. Following the Assay Kit protocol, reagent A (Malachite Green oxalate and polyvinyl alcohol) and reagent B (ammonium molybdate in 3 M sulfuric acid) were mixed at a ratio of 100:1 (*v*/*v*) immediately before use and brought to 25 °C; 25 µL of this mixture (Malachite Green working reagent) was added to 100 µL of the 5 µM NaH_2_PO_4_ solution in a 3-mm quartz cuvette and incubated for 30 min at 25 °C, as described in the manual, before measuring absorbance values over a range of 200–900 nm using a Hitachi U-3000 spectrophotometer to determine the optimal wavelength for quantifying the developed colour.

To determine the optimal temperature for colour development, the 5 µM NaH_2_PO_4_ solution was incubated with reagent A and B at 4 °C, 25 °C and 37 °C for 30 min, each, followed by monitoring the absorbance values at the optimal wavelength as determined above over a period of 60 min.

To determine the potential influence of MgCl_2_ as a possible CsaB activator on colour development, MgCl_2_ was added to a final concentration of 0, 2, 5, 10, 30 or 100 mM.

A phosphate standard curve was generated by using a 0.1 M NaH_2_PO_4_ solution with final concentrations of 0, 1, 2, 3, 6, 10, 20, 30 or 60 µM of NaH_2_PO_4_; triplicate measurements were performed at 620 nm. The measured absorbance values of the standard substance were plotted and a linear regression was calculated.

### 2.8. CsaB Activity Assay: Reaction Parameters

For assaying the activity of CsaB on its known acceptor substrate (2) [11], key reaction parameters were analysed. All measurements were performed in triplicate and obtained absorbance values were blank-corrected by a control reaction without enzyme.

Based on a previously performed one-pot reaction [25], the PEP concentration was selected as 50 µM and the acceptor (2) concentration was 150 µM, which is below the *K*_M_, but due to its difficult synthesis it was not available in larger amounts. (2) was reacted with 50 µM PEP and 0.35 µg of purified rCsaB in a 100-µL reaction volume containing 10 mM MgCl_2_ as a possible CsaB activator under different conditions of time, temperature and pH, followed by colour measurement in the 3-mm cuvette format.

The enzyme reaction was performed for 2, 5, 10 and 20 min and selected temperatures were 4 °C, 25 °C, 37 °C and 60 °C. The optimal pH was determined by performing the reaction in different buffers at a final concentration of 80 mM, including sodium citrate buffer (pH 4.0, pH 5.0), Tris-HCl (pH 6.0, pH 7.5) and Bis-Tris propane (pH 8.0, pH 9.0).

For structural confirmation of the rCsaB reaction product by NMR, the production of the pyruvylated product under optimal reaction conditions was upscaled using 1.5 mg (2).

### 2.9. Kinetic Analysis of CsaB with β-d-ManNAc-(1→4)-α-d-GlcNAc-PP-UndPh Acceptor (2)

To determine the activity of rCsaB upon variation of PEP, eight data points were generated in triplicate using reactions containing 0.35 µg CsaB and 150 µM acceptor substrate (2) in a final volume of 100 µL of 80 mM Tris-HCl, pH 7.5. The PEP concentration was varied to reach a final concentration of 2, 4, 6, 10, 30, 50, 100 and 200 µM. To evaluate the possible influence of MgCl_2_ on the enzyme velocity, the same reactions were performed with the addition of 10 mM MgCl_2_. After 5 min of incubation at 37 °C, the Malachite Green working reagent was added to stop the enzymatic reaction and the colour reaction was developed for 1 h at 37 °C followed by measuring the absorbance at 620 nm. Control reactions without enzyme were performed and data points were blank-corrected. Using the linear equation of the standard curve, the velocity was calculated by including the dilution factor of the enzyme and its concentration. Referring to the amount of rCsaB used in the assay, units (U) per minute were determined.

To estimate the activity of rCsaB upon variation of the acceptor substrate, seven data points were included, using reactions containing 0.35 µg rCsaB and 200 µM PEP (to ensure pseudo-first-order conditions) in a final volume of 100 µL of 80 mM Tris-HCl, pH 7.5. The acceptor substrate concentration was varied, reaching a final concentration of 25, 50, 75, 100, 250, 500 and 1000 µM. The enzyme reaction, measurement and calculation of velocity were performed as described above.

The data were analysed using statistical software GraphPad Prism (version 9.1.2; GraphPad, San Diego, CA, USA), where *K*_M_ and *V*_max_ values were calculated by non-linear least-square regression to the direct Michaelis–Menten plot.

### 2.10. Testing of Alternate Substrates for CsaB

To analyse the suitability of alternate CsaB substrates, rCsaB was incubated with synthesised β-d-ManNAc-(1→4)-β-d-GlcNAc-1-OMe (3) mimicking a putative CWGP repeat biosynthesis intermediate without a lipid-like tail and with *p*NP-β-d-ManNAc (5), respectively. The acceptor substrate concentration was varied, reaching a final concentration of 10 µM, 30 µM, 50 µM, 100 µM, 200 µM, 300 µM and 500 µM, each. The enzymatic reactions were carried out with 0.35 µg rCsaB and 200 µM PEP in 80 mM Tris-HCl, pH 7.5. The standard mode of measurement as described above was used.

## 3. Results

### 3.1. Expression and Purification of Recombinant Carbohydrate-Active Enzymes

To obtain ManNAc-containing acceptor substrates for the *P. alvei* CsaB enzyme, UDP-α-d-ManNAc is required. Since this compound is commercially unavailable, as an alternative to chemical synthesis, UDP-α-d-ManNAc was produced enzymatically from UDP-α-d-GlcNAc. For this purpose, the *E. coli* UDP-GlcNAc-2-epimerase WecB was produced recombinantly in *E. coli* BL21, enriched and purified using Superdex 200 SEC. In this way, rWecB (calculated molecular weight, 42.2 kDa) was obtained in high purity according to a CBB-stained 10% SDS-PAGE (Figure S1).

The inverting UDP-ManNAc transferase TagA and the ketalpyruvyltransferase CsaB from the *P. alvei* CWGP biosynthesis pathway were freshly produced as recombinant proteins and purified by use of the translationally fused MBP and His_6_-tag, respectively [11].

### 3.2. Chemical Synthesis and Purification of α-d-GlcNAc-Diphosphoryl-11-Phenoxyundecyl

To obtain α-d-GlcNAc-PP-UndPh (1), GlcNAc-1-phosphate and the 11-phenoxyundecyl phosphate portion were prepared separately and subsequently coupled together via an established methodology [24]. For the purification of (1), HILIC was employed, where the desired product was eluted in an ACN/NH_4_OAc gradient after seven column volumes.

### 3.3. Enzymatic Elongation of α-d-GlcNAc-PP-UndPh to β-d-ManNAc-α-d-GlcNAc-PP-UndPh

Two strategies were pursued for the generation of β-d-ManNAc-(1→4)-α-d-GlcNAc-PP-UndPh (2) from (1) in sufficient amounts to optimise the CsaB assay. Following strategy A, we performed an in situ coupled reaction with rWecB to epimerise UDP-α-GlcNAc to UDP-α-ManNAc, and rTagA was employed to elongate (1) to the disaccharide state (2). rWecB showed ~10% epimerisation efficiency in a 1-h/37 °C reaction, which is comparable to that obtained previously with the UDP-GlcNAc-2-epimerase MnaA from *P. alvei* [11]. Prolonged incubation (at 25 °C, overnight) of rWecB, UDP-GlcNAc and rTagA with (1) yielded full glycosylation efficiency to produce (2). Following strategy B, direct provision of an excess of synthesised UDP-α-d-ManNAc in a 1-h/37 °C rTagA reaction, followed by incubation overnight as above, reproducibly yielded (2) to completeness. The rTagA reaction towards (2) was monitored via ^1^H and ^31^P NMR spectroscopy [11].

**Figure 1:**
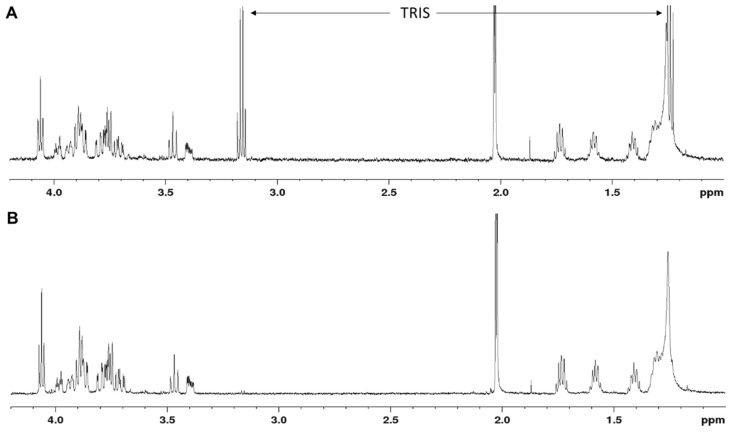
^1^H NMR of the β-d-ManNAc-(1→4)-α-d-GlcNAc-PP-UndPh acceptor before (**A**,**B**) after salt exchange with Dowex Na^+^.

Following either strategy, the reaction mixture was subjected to Sep-Pak purification [11], where (2) was recovered from the MeOH fraction as internal Tris salt in an already pure state. The Tris salt form was reflected by the presence of a characteristic multiplet at 3 ppm in the ^1^H NMR spectrum (Figure 1A). To avoid assay interference of the amino group of Tris, the Tris salt form of (2) was changed to the sodium salt via treatment with Dowex 50XW8 Na^+^ form ion exchange resin, and the removal of Tris was confirmed via ^1^H NMR, revealing the Tris signals to be absent (Figure 1B). The overall yield of purified (2) in Na^+^ form was 12 mg.

### 3.4. Chemical Synthesis of β-d-ManNAc-(1→4)-β-d-GlcNAc-1-OMe

To test if methyl-glycoside is a suitable acceptor substrate for CsaB, β-d-ManNAc-(1→4)-β-d-GlcNAc-1-OMe (3) was synthesised in 91% yield from the previously described, protected intermediate (**S1**) [26] (Supplementary Materials). ESI-TOF-MS analysis showed a mass of [M + H^+^]^+^ = 439.1927 *m/z* conforming with the theoretical mass of the target compound ([M + H^+^]^+^ = 439.1922 *m/z*). NMR data of (**3**) are shown in Table S1.

### 3.5. Set-Up of a Colorimetric CsaB Activity Assay

To optimise the colorimetric quantification of phosphate as a read-out for CsaB activity, which releases orthophosphate from the PEP substrate upon pyruvyltransfer, the Malachite Green Phosphate Assay Kit was used. Phosphate detection is based on the formation of a colour complex with Malachite Green and molybdate that is visible by a colour change from yellow-green to blue-green.

**Figure 2:**
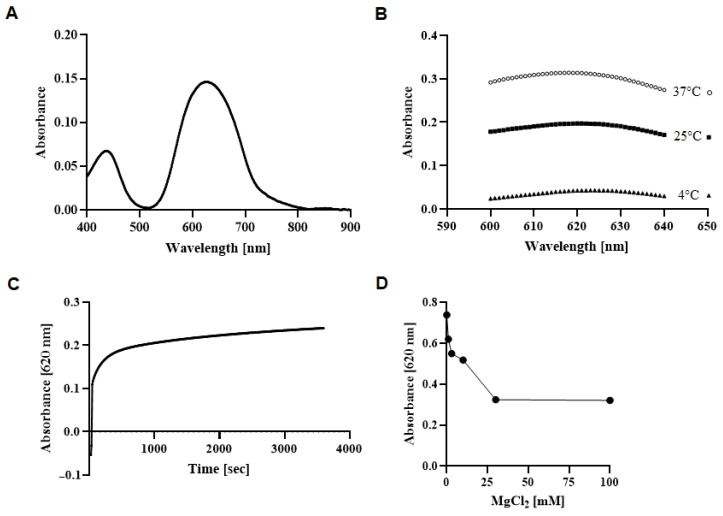
Determination of optimal parameters for colour development of a 5 µM NaH_2_PO_4_ standard solution without enzyme using the Malachite Green Phosphate Assay Kit. (**A**) Wavelength scan of the generated phosphomolybdate upon complexation with Malachite Green and molybdate. Colour development in dependence of (**B**) temperature and wavelength for measurement as well as (**C**) incubation time and (**D**) addition of MgCl_2_, with measurement at optimal wavelength of 620 nm.

A wavelength scan between 200 and 900 nm revealed the absorbance maximum of the phosphomolybdate complex to be at 620 nm (Figure 2A) and colour development being most intense after an incubation at 37 °C for 1 h (Figure 2B,C). Notably, a strong temperature dependence of the colour reaction was revealed by comparing the absorbance values at 4 °C, 25 °C and 37 °C (Figure 2B). To test if MgCl_2_ as a possible activator of CsaB interferes with colour development in the assay, several MgCl_2_ concentrations were tested. It was found that the presence of MgCl_2_ at a concentration above 2 mM reduced colour development, as evident from measuring the phosphate solution without enzyme (Figure 2D). For maximum sensitivity, colour development in the CsaB pyruvyltransferase assay was measured at 620 nm after 1 h of colour complex formation at 37 °C.

**Figure 3:**
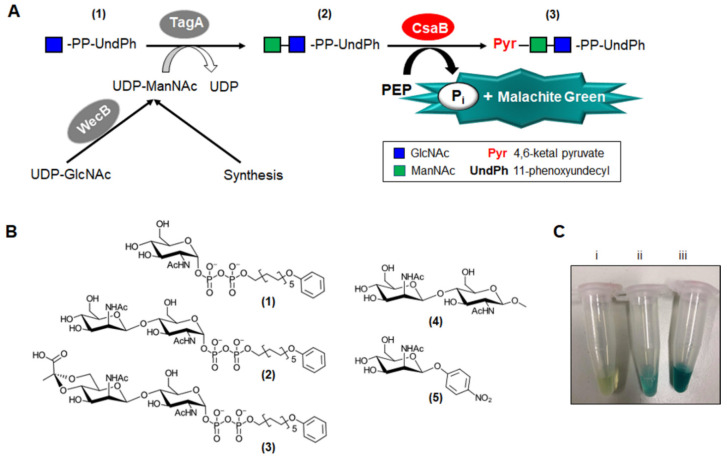
Colorimetric pyruvyltransferase activity assay based on the Malachite Green Phosphate Assay. (**A**) Chemo-enzymatic synthesis of the acceptor substrate for the 4,6-ketalpyruvyl transferase CsaB from *P. alvei* with subsequent colour reaction for assaying enzyme activity. WecB, UDP-GlcNAc-2-epimerase; TagA, ManNAc transferase. (**B**) Synthetic compounds (1), (2), (4), (5) used in this study and reaction product generated upon CsaB catalysis. (**C**) Enzyme assay using the β-d-ManNAc-(1→4)-α-d-GlcNAc-PP-UndPh acceptor (2). Reaction blank (**i**), without CsaB, 8 µM PEP, and colour development upon CsaB catalysis with varying PEP concentrations ((**ii**), CsaB, 8 µM PEP; (**iii**), CsaB, 100 µM PEP).

In an enzyme reaction set-up, where the release of phosphate from PEP and concomitant colour complex formation result from pyruvyltransfer to the saccharide acceptor (Figure 3A), 150 µM ManNAc-GlcNAc-PP-UndPh acceptor (2) (Figure 3B) and 50 µM PEP were reacted with 0.35 µg of rCsaB in buffered solution, and colour development was measured in a cuvette at 620 nm as determined above (Figure 3C).

**Figure 4:**
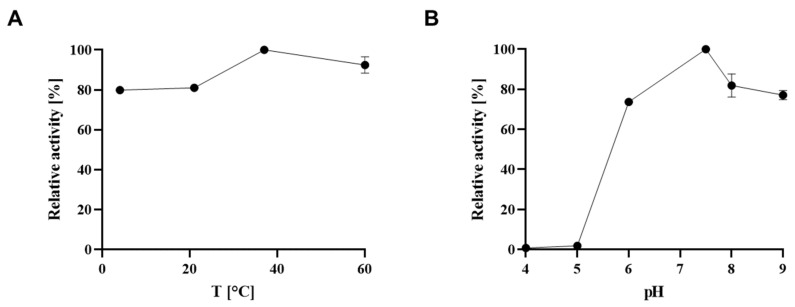
Determination of optimal conditions for the CsaB enzyme assay in regard to (**A**) temperature and (**B**) pH value. Absorbance was measured at 620 nm after 5 min of enzyme reaction and colour development for 60 min.

The optimal incubation time to catch the enzyme in the linear range (initial rate) was determined to be 5 min, with the optima of pH and temperature being between pH 6.0 and 7.5, and 37 °C (Figure 4A,B).

**Figure 5:**
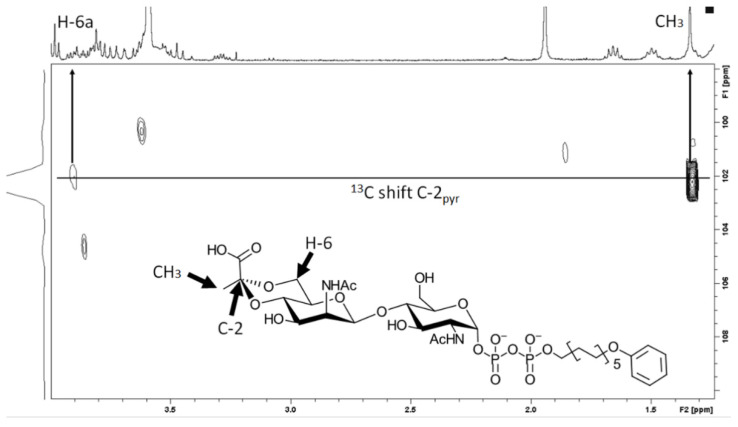
[^1^H,^13^C] Hetero multiple bond correlation (HMBC) spectra of the pyruvate C-2 region of the CsaB reaction product. The proton dimension is given in F2 while the carbon dimension is found in F1. The correlation between the pyruvate C-2 and the proton signals of the ManNAc H-6a and the pyruvate methyl group is in full agreement with [11] and shows the position of the introduced pyruvate group in acceptor (2).

NMR analysis confirmed the structure of the pyruvylated rCsaB reaction product using (2) under the conditions defined above (Figure 5).

rCsaB retained full activity over a period of four weeks when stored at a concentration of 0.4 mg mL^−1^ in Tris/HCl, pH 7.5 at 4 °C.

### 3.6. Determination of Kinetic Constants for CsaB

**Figure 6:**
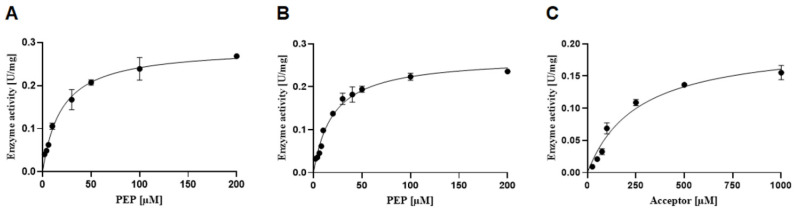
Kinetic analysis of CsaB enzyme activity revealing *K*_M_, *k*_cat_ and *k*_cat_/*K*_M_. Direct Michaelis–Menten plot for varying PEP concentration (**A**) without MgCl_2_, (**B**) with 10 mM MgCl_2_ and (**C**) upon variation of the acceptor substrate concentration with PEP fixed at 200 µM.

Kinetic analysis revealed for rCsaB a *K*_M, PEP_ value of 19.50 ± 3.50 µM, a *k*_cat, PEP_ of 0.21 ± 0.01 s^−1^ and *k*_cat_/*K*_M_ = 10.76 mM s^−1^ (Figure 6A). Next, we determined if MgCl_2_, as a suggested enzyme activator [27], affects CsaB activity. The addition of 10 mM MgCl_2_ to the pyruvyltransferase reaction did not change the *K*_M, PEP_ value (20.20 ± 2.70 µM) and marginally (but not significantly) decreased the *k*_cat, PEP_ to 0.19 ± 0.01 s^−1^ (Figure 6B). We conclude that MgCl_2_ is not necessary for CsaB activity. The *K*_M_ for GlcNAc-ManNAc-PP-UndPh was determined to be 258 ± 38 µM (*K*_M, Acceptor_) with a *k*_cat, Acceptor_ of 0.15 ± 0.01 s^−1^ and *k*_cat_/*K*_M_ = 0.58 mM s^−1^ (Figure 6C).

### 3.7. Testing Alternate Substrates for CsaB

**Figure 7:**
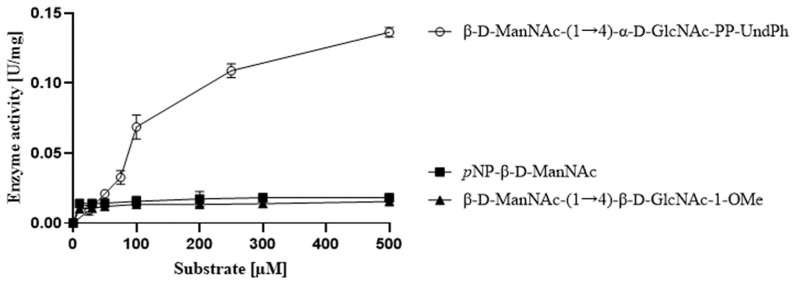
Testing *p*NP-β-d-ManNAc and β-d-ManNAc-(1→4)-β-d-GlcNAc-1-OMe as CsaB substrates under optimal assay conditions in comparison to β-d-ManNAc-(1→4)-α-d-GlcNAc-PP-UndPh. CsaB activity is given in units per mg of enzyme.

Providing 1-O Me glycoside (4) or *p*NP-β-d-ManNAc (5) as potential acceptor substrates in a kinetics CsaB reaction set-up without MgCl_2_, the detectable colour development did not exceed that of the control reaction without enzyme (Figure 7). This implies that neither of these compounds is a suitable acceptor substrate for *P. alvei* CsaB.

## 4. Discussion

Pyruvyltransferases are widespread in nature; they occur in almost all bacterial phyla, several yeast species and in algae, but not in humans [1]. They catalyse pyruvate formation as a biologically potent non-carbohydrate modification of various glycoconjugates [1] and are promising anti-infective targets. The enol-pyruvyltransferase MurA from the bacterial peptidoglycan biosynthesis pathway, for instance, imparts fosfomycin resistance and is currently under evaluation towards new inhibitors [19]. According to the World Health Organization, 750,000 deaths per year are caused by antibiotic-resistant bacteria, and the rise of antibiotic resistances necessitates alternate strategies to counteract bacterial infections. Traditionally, the bacterial cell wall is a prominent target point for antimicrobial agents. For instance, one Achilles heel of the methicillin-resistant superbug *Staphylococcus aureus* is its cell wall teichoic acid; if enzymes within its biosynthesis pathway are disrupted, β-lactam antibiotic sensitivity is restored and host colonisation is impaired [28,29].

A 4,6-ketalpyruvylated β-d-ManNAc residue as an integral part of various bacterial CWGPs is crucial for sticking the Gram-positive cell wall together [6,9,30]. *B. anthracis* is the most prominent example of a pathogen that has integrated the pyr-ManNAc epitope into its cell wall building plan [9]. To uncover details of ManNAc pyruvylation, the honeybee saprophyte *P. alvei* serves as an ideal model due to analogies of CWGP composition and overall cell wall architecture with *B. anthracis*. We have previously identified the CsaB enzyme encoded in the *P. alvei* CWGP biosynthesis gene cluster as a 4,6-ketalpyruvyl::ManNAc transferase that is active on the synthetic CWGP biosynthesis precursor analogue β-d-ManNAc-(1→4)-α-d-GlcNAc-PP-UndPh (2) [11]. A CsaB homologue is also encoded in the *B. anthracis* CWGP biosynthesis gene locus [31].

Currently, there is no fast and straightforward assay for the measurement of the activity and kinetics of pyruvyltransferases available. To circumvent the requirement of labelled acceptor substrates for on-column detection and isolation of pyruvylated reaction products, the Malachite Green Phosphate Assay [18,19,20,21] was optimised for assaying ketalpyruvyltransfer to synthetic saccharide acceptor substrates, exemplified with *P. alvei* CsaB. The assay with a sensitivity range of 0.02 to 40 µM phosphate (according to the manufacturer) is based on phosphate release during the pyruvyltransfer reaction due to the splitting of PEP into a pyruvate entity and inorganic phosphate followed by a colour reaction. Notably, absorbance values of the formed colour complex measured in microtiter plates in the Tecan plate reader were generally lower and noisier than those measured spectrophotometrically in a 3-mm quartz cuvette (F.F. Hager-Mair, C. Stefanović, data not shown). For this reason, the enzyme assay was optimised for the cuvette format. In the colour reaction, we obtained high background values in control reactions without enzyme, using β-d-ManNAc-(1→4)-α-d-GlcNAc-PP-UndPh (2) as a CsaB acceptor substrate, which we initially attributed to the complexity of the lipid-like tail of (2); lipids were described in the Sigma-Aldrich manual to interfere with the Malachite Green dye, with the chemistry behind it unknown. Surprisingly, the use of methanol, ethanol or propanol in a control reaction yielded a comparably high background signal. Thus, it is imperative to perform the full range of control reactions when assaying pyruvyltransferases with the Malachite Green Phosphate Assay in order to avoid false positive results. Furthermore, given the strong temperature dependence of the colour reaction (Figure 2B), the temperature should be tightly controlled to obtain reliable results. Notably, these findings might also be of relevance when using the Malachite Green Assay Kit for investigating other enzymes that release inorganic phosphate.

As defined within this study, an optimal assay set-up for quantifying phosphate release upon pyruvyltransfer catalysed by *P. alvei* CsaB contains 0.35 µg of recombinant enzyme, 200 µM PEP and an acceptor concentration above *K*_M_ (250–1000 µM, if enough acceptor is available), with colour development for 60 min and measurement in a 3-mm quartz cuvette at 620 nm. Notably, a shortage of synthesised acceptor (2) precluded its use at a concentration ensuring a saturating concentration in the assays. rCsaB was determined to have optimal activity at 37 °C and pH 7.5. The addition of MgCl_2_ to the assay had no significant effect on the catalytic activity of *P. alvei* CsaB (Figure 6A,B). Notably, MgCl_2_ above a concentration of 2 mM seems to decrease colour development in the Malachite Green Phosphate Assay under the chosen conditions, possibly due to interference with assay reagents or phosphate or PEP (compare with Figure 2D) [32]. Notably, for the yeast pyruvyltransferase Pvg1p, an inhibitory effect of Co^2+^, Ni^2+^ and Cd^2+^ was reported [15]; in that study, however, Mg^2+^ was not included.

Using the reaction set-up defined within the frame of this study, kinetic constants could be determined for rCsaB by fitting the data to the Michaelis–Menten equation (Table 1). This revealed a *K*_M_ value for the PEP donor substrate of 19.50 ± 3.5 µM, which is ~10-fold lower compared to the *K*_M, PEP_ reported for the *B. fragilis* CPS 4,6-ketalpyruvl::galactose transferase WcfO [4], suggesting a higher affinity of CsaB for the donor substrate. Of note, *K*_M, PEP_ values of 199 µM, 121 µM and of 0.4 µM were reported for two mycobacterial UDP-*N*-acetylglucosamine enolpyruvyl transferases (*M. tuberculosis* MurA and *M. smegmatis* MurA) and for *E. coli* MurA, respectively [18,33]. Furthermore, our study presents the first kinetic constants of a pyruvyltransferase towards the acceptor substrate, with *K*_M, Acceptor_ = 258.00 ± 38.00 µM and a *k*_cat, Acceptor_ of 0.15 ± 0.01 s^−1^ as determined for CsaB towards β-d-ManNAc-(1→4)-α-d-GlcNAc-PP-UndPh (2). The *K*_M, Acceptor_ is ~13 times higher than the *K*_M, PEP_, indicating that the affinity of CsaB for PEP is higher than for the acceptor.

The failure of CsaB to catalyse ketalpyruvyltransfer to both *p*NP-β-d-ManNAc and β-d-ManNAc-(1→4)-β-d-GlcNAc-1-OMe (3) supports the necessity of a lipid-like tail and/or phosphate on a suitable CsaB substrate. This assumption is in agreement with data obtained with *B. fragilis* WcfO, which was found to be active on a lipid-bound tetrasaccharide CPS repeat [4], and with the predicted CWGP acceptor substrate for *B. anthracis* CsaB [31].

The developed enzyme assay is crucial for future mechanistic studies by the use of rationally designed pyruvyltransferases as well as for future inhibitor design to combat bacterial pathogens.

## Figures and Tables

**Table 1:** Kinetic constants of *P. alvei* CsaB in comparison to other pyruvyltransferases. (2), β-d-ManNAc-(1→4)-α-d-GlcNAc-PP-UndPh; (*), 2AA-B(6Z)PP-AADGal-Gal (2AA-B(6Z)PP, 2-nitrileanilino-bactoprenyl diphosphate; α-d-2-*N*-acetylamido-4-amino-galactopyranose); n.d., not determined.

Enzyme	Substrate	*K*_M_ (µM)	*k*_cat_ (s^−1^)	*k*_cat_/*K*_M_ (mM s^−1^)	Reference
*P. alvei* CsaB (Ketalpyruvyltransferase)	PEP Acceptor (2)	19.50 ± 3.50 258.00 ± 38.00	0.21 ± 0.01 0.15 ± 0.01	10.76 0.58	This study
*B. fragilis* WcfO (Ketalpyruvyltransferase)	PEP Acceptor (*)	299 ± 49 n.d.	0.249 ± 0.01 n.d.	0.83	[4]
*M. tuberculosis* MurA (Enolpyruvyltransferase)	PEP UDP-GlcNAc	199 ± 13 2743 ± 231	0.058 ± 0.00 0.033 ± 0.00	0.29 0.01	[18]
*M. smegmatis* MurA (Enolpyruvyltransferase)	PEP UDP-GlcNAc	121 ± 80 2320 ± 800	0.117 ± 0.01 0.147 ± 0.01	0.97 0.06	[18]
*E. coli* MurA (Enolpyruvyltransferase)	PEP UDP-GlcNAc	0.4 15.0	3.8 3.8	9.50 0.25	[33]

## Data Availability

The data presented in this study are available in the Supplementary Materials or upon request from C.S. (Christina Schäffer).

## Supplementary Materials

The following are available online at https://www.mdpi.com/article/10.3390/biom11111732/s1, Supplementary Methods: Chemical synthesis of β-d-ManNAc-(1→4)-β-d-GlcNAc-1-OMe (**3**). Figure S1: SDS-PAGE analysis of purified, recombinant UDP-GlcNAc-2-epimerase WecB (5 µg; calculated molecular weight, 42.2 kDa) from *E. coli* run on a 10% SDS-PAGE gel and visualised with Coomassie Brilliant Blue G250 staining (WecB). Molecular weight standard (MW), PageRuler Prestained Plus Protein Ladder (Thermofisher). Table S1: ^1^H and ^13^C chemical shifts (δ ppm) and in parentheses *J* couplings (Hz) for β-d-ManNAc-(1→4)-β-d-GlcNAc-1-OMe (**3**).
