# Supplementary material for: Assaying Paenibacillus alvei CsaB-Catalysed Ketalpyruvyltransfer to Saccharides by Measurement of Phosphate Release
Source: Biomolecules. 2021 Nov 20;11(11):1732. doi: 10.3390/biom11111732 (PMC8615578; doi:10.3390/biom11111732)
Supplement: Supplementary file 1 [file biomolecules-11-01732-s001.zip › biomolecules-1447042-supplementary.pdf]

biomolecules

MDPI

Supplementary Information

Assaying *Paenibacillus alvei* CsaB-Catalysed Ketalpyruvyltransfer to Saccharides by Measurement of Phosphate Release

Fiona F. Hager-Mair[1,#], Cordula Stefanović[1,#], Charlie Lim[2], Katharina Webhofer[2], Simon Krauter[2], Markus Blaukopf[2], Roland Ludwig[3], Paul Kosma[2], and Christina Schäffer[1,*]

[1] *NanoGlycobiology* Unit, Department of NanoBiotechnology, Universität für Bodenkultur Wien, 1190 Vienna, Austria; fiona.hager@boku.ac.at (F.F.H.-M.); cordula.stefanovic@boku.ac.at (C.S.); christina schaeffer@boku.ac.at (Christina Schäffer)
[2] Department of Chemistry, Institute of Organic Chemistry, Universität für Bodenkultur Wien, 1190 Vienna, Austria; charlie.lim@boku.ac.at (C.L.); katharina.webhofer.20@ucl.ac.uk (K.W.); simon@krauter.at (S.K.); markus.blaukopf@boku.ac.at (M.B.), paul.kosma@boku.ac.at (P.K.)
[3] Biocatalysis and Biosensing Laboratory, Department of Food Science and Technology, Universität für Bodenkultur Wien, 1190 Vienna, Austria; roland.ludwig@boku.ac.at
* Correspondence: christina.schaeffer@boku.ac.at; Tel.: +43-1-47654 (ext. 80203)

* Correspondence: christina.schaeffer@boku.ac.at; Tel.: +43-1-47654 ext. 80203
\# These authors contributed equally

Supplementary Methods

*Chemical Synthesis of β-D-ManNAc-(1→4)-β-D-GlcNAc-1-OMe (**3**)*

To synthesize β-D-ManNAc-(1→4)-β-D-GlcNAc-1-OMe (**3**), methyl 2′-acetamido-3′-O-benzyl-4′,6′-O-benzylidene-2′-deoxy-β-D-mannopyranosyl-(1→4)-2-acetamido-3,6-di-O-benzyl-2-deoxy-β-D-glucopyranoside **S1** (6 mg; 7 μmol) - an intermediate from the synthesis of the [→4)-β-D-GlcNAc-(1→3)-4,6-Pyr-β-D-ManNAc-(1→] repeat of the *P. alvei* CWGP [1] - was dissolved in dry MeOH (1.5 ml), a catalytic amount of Pd/C was added, and the suspension was flushed with argon. The flask was evacuated, flushed with Ar four times, evacuated again, and set under H_2-atmosphere. After 5.25 h of reaction time, the catalyst was filtered over Celite® and washed with MeOH several times (15 ml in total). The filtrate was concentrated *in vacuo* to give a crude residue (~4 mg). NMR analysis showed a mixture of (**3**) and the benzylidene-protected intermediate (Scheme S1). Therefore, the reaction was repeated overnight, finally yielding 3 mg (91%) of (**3**) as an amorphous solid.

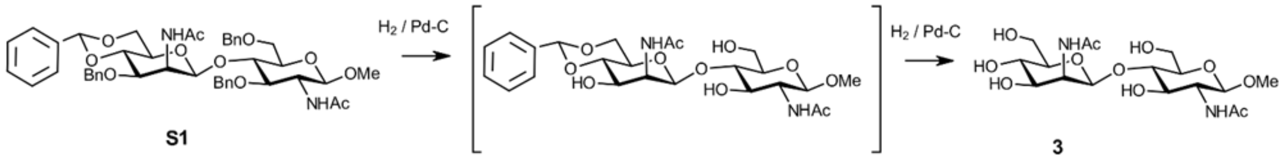

Scheme S1. Synthesis of β-D-ManNAc-(1→4)-β-D-GlcNAc-1-OMe (**3**).

1. Krauter, S.; Schäffer, C.; Kosma, P. Synthesis of a pyruvylated *N*-acetyl-β-D-mannosamine containing disaccharide repeating unit of a cell wall glycopolymer from *Paenibacillus alvei Arkivoc* **2021**, 137-151. doi:10.24820/ark.5550190.p011.358

Supplementary Figure S1

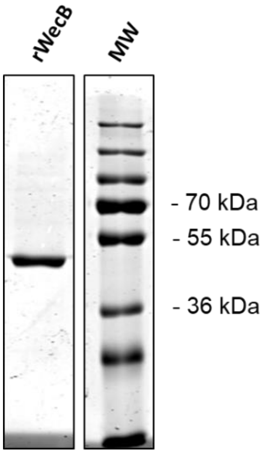

Figure S1. SDS-PAGE analysis of purified, recombinant UDP-GlcNAc-2-epimerase WecB (5 µg; calculated molecular weight, 42.2 kDa) from *E. coli* run on a 10% SDS-PAGE gel and visualized with Coomassie Brilliant Blue G250 staining (rWecB). Molecular weight standard (MW), PageRuler Prestained Plus Protein Ladder (Thermofisher).

Supplementary Table S1

Table S1. ^{1}H and ^{13}C chemical shifts (δ, ppm) and in parentheses *J* couplings (Hz) for β-D-ManNAc-(1→4)-β-D-GlcNAc-1-OMe (**3**).

**Sugar residue**	**β-D-ManNAc-(1→**	**→4)-β-D-GlcNAc-1-OMe**
H1	4.88 (1.6)	4.42 (8.0)
C1	99.41	101.94
H2	4.54 (1.6, 4.6)	~3.70 (n.d.)
C2	53.24	55.16
H3	3.81 (4.4, 9.7)	~3.70 (n.d.)
C3	71.96	72.47
H4	~3.50 (n.d)	~3.70 (n.d.)
C4	66.62	78.87
H5	~3.50 (n.d.)	3.43 (2.7, 5.0, 9.9)
C5	74.52	76.53
H6	a: 3.87 (2.4, 12.3)	a:3.89 (2.3, 12.6)
	b: ~ 3.70 (n.d.)	b: 3.79 (5.3, 12.2)
C6	60.18	60.41
NCOC*H*$_3$	2.05 or 2.02	2.05 or 2.02
NCOCH$_3$	22.21 or 22.01	22.21 or 22.01
NCOCH$_3$	175.47 or 174.76	175.47 or 174.76
OC*H*$_3$		3.48
OCH$_3$		57.14
